# Case Report: A case study of perianal paget’s disease in a patient receiving adjuvant photodynamic therapy post-surgery

**DOI:** 10.3389/fsurg.2025.1634742

**Published:** 2025-10-08

**Authors:** Dexin Wang, Lei Liang, Jingyi Zhang, Xiaohe Zhang, Xiubi Zhang, Gang Tian, Weiliang Du, Chaochi Yue

**Affiliations:** 1 College of Integrative Chinese and Western Medicine, Southwest Medical University, Luzhou, Sichuan, China; 2 Mianyang 404 Hospital, Mianyang, Sichuan, China; 3 Department of Laboratory Medicine, The Affiliated Hospital of Southwest Medical University, Luzhou, Sichuan, China; 4 Department of Traditional Chinese Medicine, The Affiliated Hospital of Southwest Medical University, Luzhou, Sichuan, China

**Keywords:** case report, perianal paget's disease, photodynamic therapy, surgery, therapy

## Abstract

**Background:**

Extramammary Paget's disease (EMPD) is a rare malignant skin cancer, particularly uncommon in the vulvar and perianal regions, with Perianal Paget's disease (PPD) being an even rarer subtype. Due to its obscure onset location, atypical clinical manifestations, and lack of standardized treatment protocols, PPD poses significant challenges in clinical practice.

**Case presentation:**

Here, we report a case of a 61-year-old female who presented with a perianal lump discovered a year ago, which had progressively increased in size over the past four months. Imaging and endoscopic examinations revealed no additional tumors elsewhere. The patient underwent Wide local excision surgery with skin flap reconstruction. Histopathological analysis confirmed PPD. After two months from the surgery, the patient received a total of three Photodynamic therapy (PDT). Following treatment, the patient experienced mild pain, which was effectively managed. The flap is currently functioning well, with regular patient monitoring showing no signs of recurrence or metastasis.

**Conclusion:**

PDT exhibits bactericidal, anti-inflammatory, circulatory enhancement, and antitumor properties, which play a beneficial role in the treatment of PPD. This can aid in standardizing the subsequent treatment protocols for PPD.

## Introduction

Extramammary Paget's disease (EMPD) is a rare malignant tumor that typically affects apocrine gland-bearing regions such as the vulva and perianal area. The reported annual incidence ranges from 0.1 to 2.4 cases per 1,000,000 individuals, predominantly occurring in the elderly (1). Clinically, EMPD presents with localized skin manifestations including erythema, pruritus, and scaling, while a minority of patients may remain asymptomatic. EMPD is commonly classified into primary cutaneous origin and secondary cutaneous origin (2). The pathogenesis of EMPD remains unclear, with the prevailing view suggesting that primary EMPD primarily originates from apocrine gland carcinoma (3). Secondary EMPD is typically caused by malignancies of the rectum or urogenital tract, leading to a poor prognosis. Therefore, additional diagnostic tests such as Computed Tomography scans and ultrasound are often necessary to assess and confirm the presence of potential cancers in other organs (4).

Perianal Paget's disease (PPD) is relatively rare, accounting for approximately 20% of EMPD cases (5). While surgical resection and radical surgery remain key strategies for treating PPD, a standardized treatment protocol has not yet been established (1). Currently, surgical options include wide local excision (WLE) and Mohs micrographic surgery (MMS). Due to its relatively shorter duration and lower cost compared to MMS, WLE has long been considered the primary treatment for PPD, despite its higher recurrence rate (2, 6). Therefore, in addition to surgical intervention, alternative adjunct therapies such as photodynamic therapy (PDT), CO₂ laser ablation, radiation therapy, and chemotherapy are currently available (7). Within this study, we delineate an unusual case of primary PPD that is asymptomatic and lacks cytokeratin-7 (CK-7) expression. Due to the lack of clear clinical symptoms in patients, the initial diagnosis is often challenging. Our treatment strategy involves the adjunctive use of PDT following WLE surgery, leveraging the advantages of non-invasiveness and high efficacy, which led to demonstrating good flap function and favorable clinical outcomes that merit reporting.

## Overview of patient’s medical history

**Figure 1:**
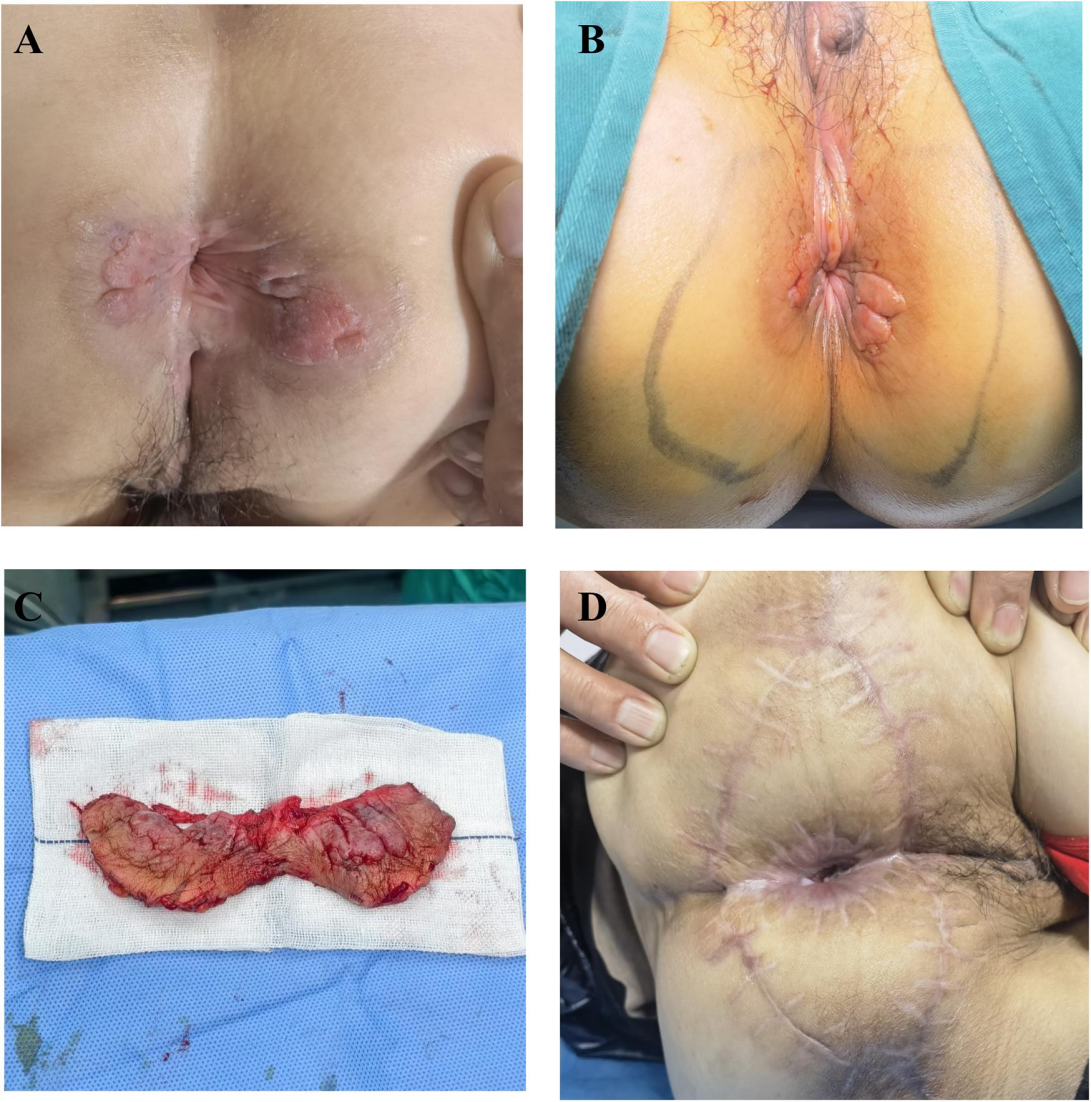
**(A)** preoperative assessment of the patient's physical condition. **(B)** Surgical resection extent. **(C)** The excised pathological tissue. **(D)** The postoperative wound recovery status at 4 months.

**Figure 2:**
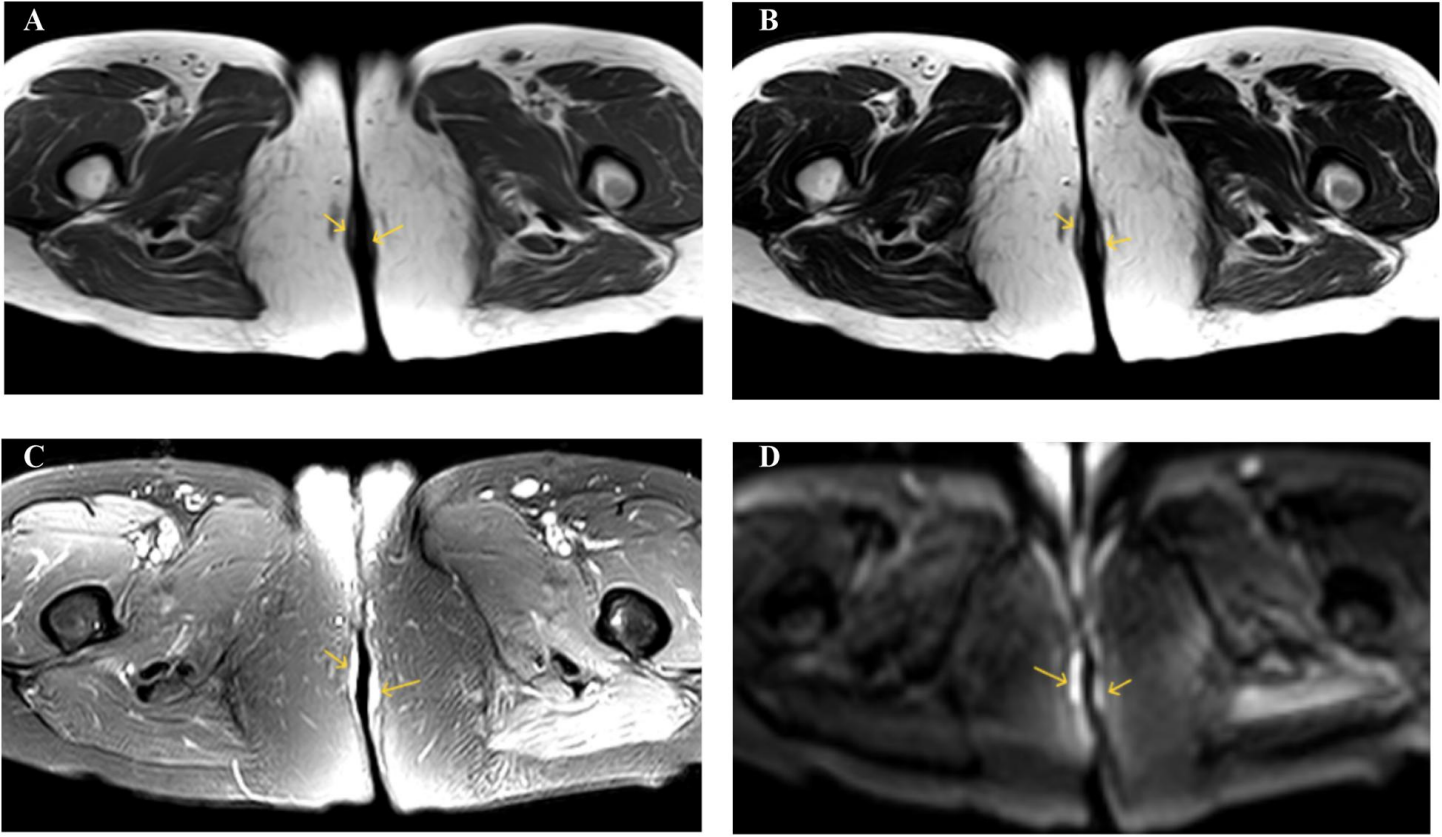
**(A)** T1WI shows low signal intensity. **(B)** T2WI shows high signal intensity. **(C)** SPAIR shows high signal intensity. **(D)** DWI shows high signal intensity.

The 61-year-old female patient was admitted to the hospital due to the discovery of a perianal mass persisting for over a year, with self-reported enlargement of the lump for more than four months. The patient presented without evident clinical symptoms such as itching or scaling, with no history of specific medical conditions or prior relevant treatment. We conducted a thorough physical examination of the patients in the inpatient department, inquired about their infectious disease history, and ruled out genital warts, syphilis, and other hospitalization records. During the physical examination, bilateral perianal masses measuring approximately 3 × 3 cm were observed. These masses appeared red in color and elicited tenderness upon palpation (Figure 1A). During hospitalization, the patient underwent colonoscopy, contrast-enhanced CT of the abdomen, and contrast-enhanced Magnetic Resonance Imaging of the pelvis to rule out metastasis to other sites. Colonoscopy revealed a colonic polyp measuring approximately 0.3 × 0.4 cm, with no evidence of tumors (Supplementary Figure S1). Abdominal CT scan showed no abnormalities. The lesion in the pelvic MRI appears as low signal on T1-weighted imaging, high signal on T2-weighted imaging and diffusion-weighted imaging, and demonstrates significant enhancement (Figure 2). When tumor markers fall within normal ranges and are combined with immunohistochemistry results, other neoplastic conditions are ruled out, confirming the patient's primary PPD.

## Surgery and follow-up

**Figure 3:**
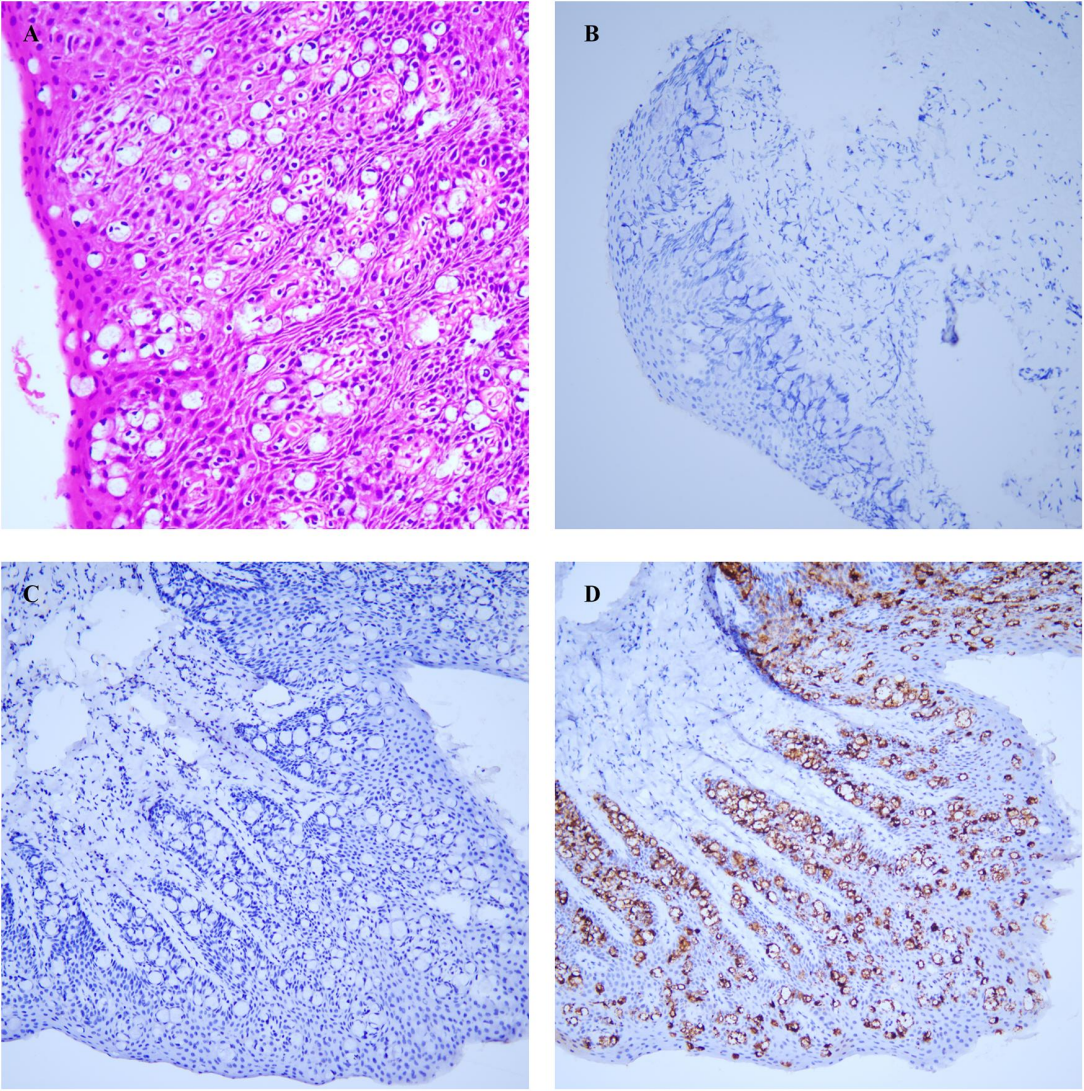
**(A)** atypical cells were identified in the squamous epithelium. **(B)** CK-7. **(C)** CK-20. **(D)** MUC-2. All photos were captured using an optical microscope at a magnification of 100 times.

We performed an excision of perianal masses on the patient concurrently with a skin flap transplantation procedure. We excised the entire lesion with a 2 cm margin beyond the lesion edge, including a 1 cm subcutaneous fat layer. Following skin lesion removal, a V–Y advancement flap of the gluteal muscle was employed for transplantation. The excised perianal lesion is shown in Figures 1B,C. The surgical procedure proceeded smoothly. On the first postoperative day, the patient exhibited slight bleeding and mild swelling in the skin flap, which resolved after the third day. Neurological function was assessed one week later, revealing no tumor recurrence, anal stenosis, anal mucosa prolapse, or wound dehiscence. Histopathological examination revealed pseudoepitheliomatous hyperplasia of squamous epithelium with suspicious atypia and mucin secretion (Figure 3A). Immunohistochemistry showed negative for CK-7, cytokeratin-20 (CK-20), caudal type homeobox 2 (CDX2), and gross cystic disease fluid protein-15 (GCDFP-15) were negative. Mucin-2 (MUC-2) is positive (Figures 3B–D). Histopathology meets the diagnostic criteria for PPD. The patient underwent their first PDT adjuvant therapy over two months after WLE. Subsequently, PDT was performed every two weeks for a total of three treatments. Using methyl aminolevulinate (M-ALA) as the photosensitizer, approximately 1 g is applied around the lesion at a distance of 1 cm each time. After 3 h of incubation, rinse with 0.9% saline solution and expose the treated area to red light at 620 nm (37 J/cm^2^) for 10 min. After irradiation, the patient experience mild pain at the irradiated site, which we alleviate by employing medical dressings and administering oral nonsteroidal anti-inflammatory drugs.

**Figure 4:**
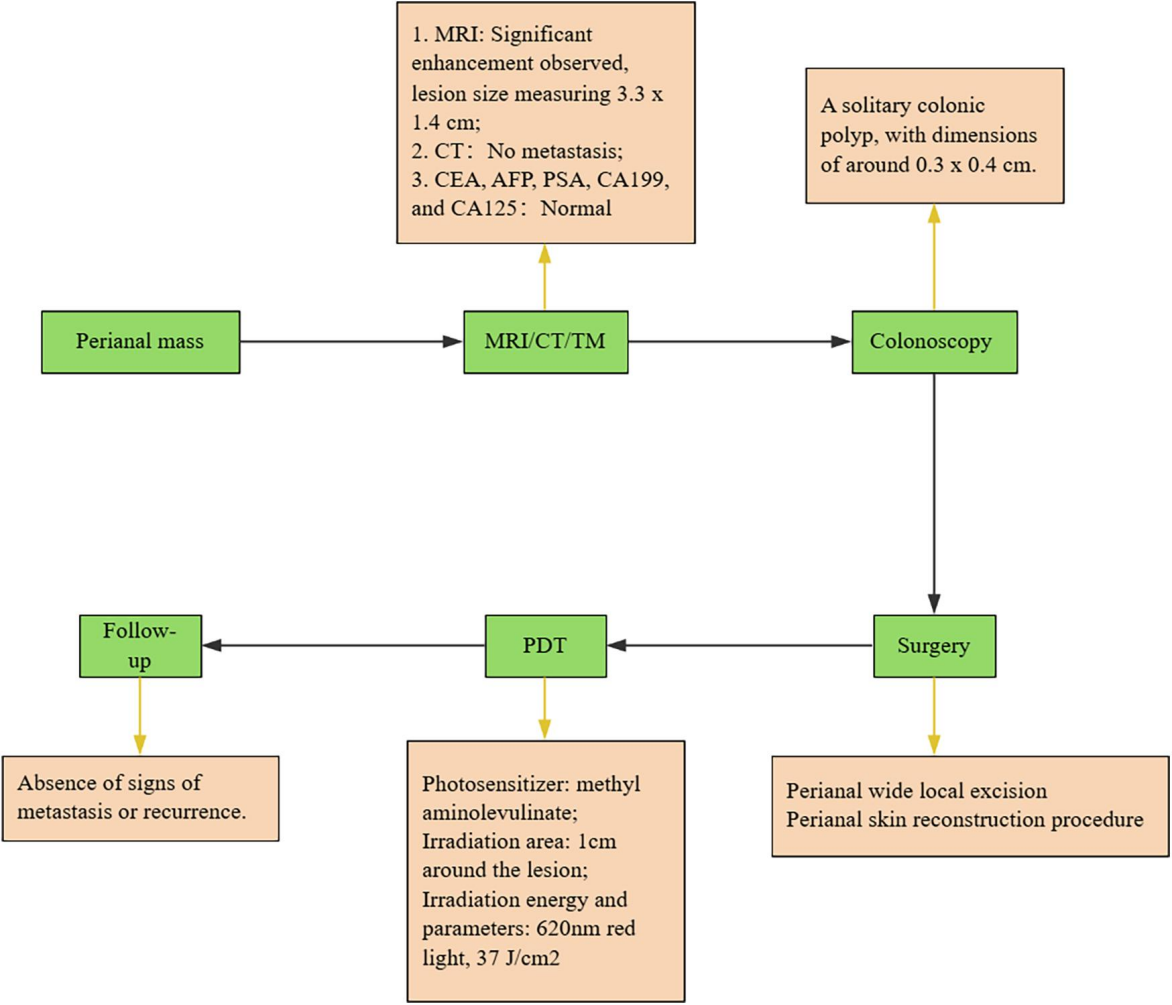
Overview of the treatment process. All green steps are presented in chronological order. The yellow sections provide explanations for each step. Carcinoembryonic antigen, CEA; Alpha fetoprotein, AFP; Carbohydrate antigen199, CA199; Tumor markers, TM.

We conduct outpatient follow-up visits with the patients. Two months post PDT, the patient remains in stable condition with normal bowel movements, well-functioning skin flap, and absence of significant clinical symptoms. The patient expressed satisfaction with the outcome. Postoperative adjuvant PDT therapy has been proven to effectively improve patient prognosis and quality of life, particularly in the successful restoration of local skin tissue and anal function. Upon reevaluation, no abnormal elevation in tumor markers was detected. This is attributed to photodynamic therapy, which destroys target tissue while preserving healthy tissue, also exhibiting antimicrobial and anti-inflammatory properties, promoting the restoration of flap function. The chronological sequence of the patient's diagnosis and treatment is depicted in Figure 4.

## Discussion

Due to the lack of specificity in the symptoms of EMPD and the inconspicuous location of its onset, diagnosis is often challenging. Easily confused skin conditions include eczema, Bowen's disease, and psoriasis, which are primarily differentiated through pathological examination. Anal eczema manifests as inflammation of the skin (8). Bowen's disease pathology reveals atypicality in all layers of the epidermal cells (9). Psoriasis is characterized by epidermal hyperplasia and elongation of the epidermal papillae (10). EMPD typically exhibits the presence of Paget cells, which are large atypical cells with abundant clear cytoplasm. For an accurate diagnosis of primary and secondary EMPD, a thorough histopathological examination utilizing different immunohistochemical markers is essential, given the varied treatment strategies and prognoses. GCDFP-15 plays a crucial role in distinguishing secondary EMPD. This protein is typically negative in primary EMPD (11). CK7 and CK20 staining can also be utilized for distinguishing primary EMPD from secondary EMPD. Primary EMPD typically exhibits CK7+/CK20- expression, while secondary EMPD arising from gastrointestinal tumors usually shows CK7-/CK20+ expression (6). However, cases of CK7-/CK20+ primary EMPD and CK7+/CK20- secondary EMPD have also been reported (12, 13). Additionally, EMPD secondary to urothelial tissue commonly presents as CK7+/CK20+ (14). It is noteworthy that a case report demonstrated CK20 negativity in secondary EMPD originating from urothelial tissue (15). The reliability of CK-7 and CK-20 in distinguishing between primary and secondary remains uncertain. In this report, the patient exhibited CK7 negativity. The specific origin and molecular mechanisms of CK7-negative Paget cells remain unclear. Despite primary EMPD typically showing strong positive CK-7 expression, however, reports do exist of primary EMPD patients who are CK7-negative (16). Another marker, CDX-2, can be utilized for discrimination as it is usually negative in primary EMPD.

In this study, the patient was CK-7 negative, but when combined with other proteins and relevant auxiliary tests (such as imaging examinations, tumor markers), we can temporarily rule out tumors from other sites. Therefore, CK-7 negativity alone cannot completely exclude the possibility of the patient having primary EMPD, usually requiring the expression of other markers. In conclusion, a comprehensive assessment using multiple markers is necessary to differentiate primary EMPD from secondary EMPD (1, 4, 6).

Surgical treatment is crucial for EMPD, with options including WLE, and MMS. The WLE has long been regarded as the standard for managing EMPD, typically with a 2 cm margin. A study indicates a high recurrence rate of 60% post WLE (17). The difficulty in determining the extent of surgical excision may stem from the deeper invasion of Paget cells, with tumor infiltration potentially preceding visible changes in the skin lesion, yet studies suggest that the choice of surgical margins (≥2 cm or ≤2 cm) is not correlated with local recurrence (18). In addition to recurrence rates, another challenge of WLE is the reconstruction of extensive tissue defects, necessitating flap surgery. The reconstruction of the perianal region must ensure both functionality and aesthetics, commonly achieved through the use of thigh-based flaps known for their ease of mobilization and high survival rates (19). A study reported on three cases of perianal reconstruction using thigh-based flaps, yielding satisfactory outcomes (20). Our patient, reconstructed with a V–Y advancement flap of the gluteal muscle, currently exhibits excellent flap functionality recovery. While WLE remains the preferred surgical method, MMS is gaining increasing attention due to its low recurrence rate. However, due to the extensive skin lesions typically seen in EMPD, the MMS approach is more time-consuming and costly.

Currently, treating PPD poses a challenge as there is a lack of standardized treatment guidelines, resulting in various available methods such as PDT, CO₂ laser ablation, radiation therapy, and chemotherapy. PDT is a non-invasive treatment method utilizing photosensitive drugs. Upon absorption of the drug by tumor cells, exposure to light of a specific wavelength induces the generation of toxic free radicals, leading to cell destruction. The anti-tumor effects of PDT are beneficial in alleviating EMPD-related symptoms (6, 21). PDT exhibits bactericidal effects on common perianal pathogens such as Staphylococcus aureus, reducing postoperative infections and enhancing flap functionality restoration (22). PDT can enhance the expression of vascular endothelial growth factor to improve blood supply to the transplanted skin flap. However, it may also potentially contribute to tumor recurrence, necessitating combined treatment with anti-angiogenic agents (23). Due to the low toxicity and repeatability of PDT, it is beneficial for long-term relief in PPD patients. In this case, following PDT treatment, the patient presented with pain only, without exhibiting severe photosensitivity. The skin flap exhibited a color similar to the surrounding skin, with a soft and elastic texture.

Due to the high recurrence rate of EMPD, regular histological examinations, such as skin biopsies, can help identify local recurrences or residual lesions early. The histological features, such as depth of invasion (e.g., stromal invasion or lymphatic spread), are directly associated with the risk of recurrence. Monitoring these indicators during follow-up aids in predicting disease progression. Additionally, histological testing is used to confirm treatment response and assess prognosis. Therefore, any eczema-like lesions appearing at the surgical site or elsewhere postoperatively should be biopsied promptly to assess the recurrence situation.

In conclusion, surgery is typically the primary treatment for EMPD, although postoperative recurrence rates are high, often necessitating adjuvant therapies. PDT offers advantages of low toxicity, minimal impact on normal tissues, and more thorough treatment, and has been utilized for EMPD. However, current research on the combination of surgery and PDT for EMPD mainly consists of retrospective analyses or case reports, lacking sufficient evidence. Therefore, future studies should aim for large-sample, multicenter, randomized controlled trials to further validate the efficacy of this combined therapy.

Furthermore, the current study has certain limitations. Firstly, it reported survival outcomes only up to nearly 4 months post-surgery, with a relatively short clinical follow-up period. Future investigations should involve longer-term monitoring to assess patients’ extended survival prognosis. Subsequently, following PDT treatment, there was no biopsy performed on the subsequent perianal skin.

## Data Availability

The original contributions presented in the study are included in the article/Supplementary Material, further inquiries can be directed to the corresponding author.

## Supplementary material

Supplementary Figure S1 Colonoscopy showing colonic polyps indicated by black arrows.
