# Supplementary figures and images for: Case Report: A case study of perianal paget’s disease in a patient receiving adjuvant photodynamic therapy post-surgery
Source: Front Surg. 2025 Oct 8;12:1634742. doi: 10.3389/fsurg.2025.1634742 (PMC12540366; doi:10.3389/fsurg.2025.1634742)

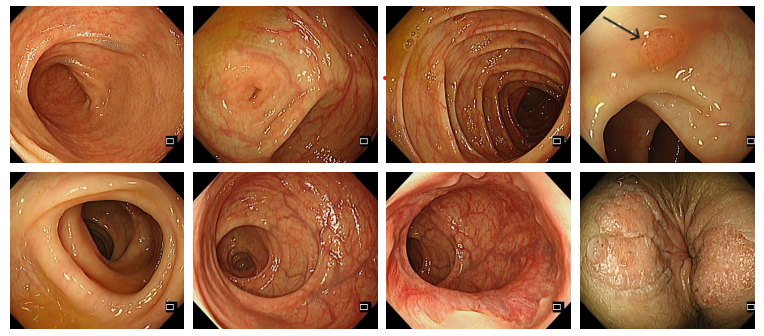

Supplement: Supplementary Figure S1 — Colonoscopy showing colonic polyps indicated by black arrows. [file Image1.tif]
